# Whole-Genome Sequencing Investigation of a Large Nosocomial Outbreak Caused by ST131 H30Rx KPC-Producing *Escherichia coli* in Italy

**DOI:** 10.3390/antibiotics10060718

**Published:** 2021-06-15

**Authors:** Aurora Piazza, Luigi Principe, Francesco Comandatore, Matteo Perini, Elisa Meroni, Vittoria Mattioni Marchetti, Roberta Migliavacca, Francesco Luzzaro

**Affiliations:** 1 Department of Clinical-Surgical, Diagnostic and Pediatric Sciences, Unit of Microbiology and Clinical Microbiology, University of Pavia, 27100 Pavia, Italy; roberta.migliavacca@unipv.it; 2 Clinical Pathology and Microbiology Unit, S. Giovanni di Dio Hospital, 88900 Crotone, Italy; luigi.principe@gmail.com; 3 Romeo and Enrica Invernizzi Pediatric Research Center, Department of Biomedical and Clinical Sciences L. Sacco, University of Milan, 20157 Milan, Italy; francesco.comandatore@unimi.it (F.C.); matteo.perini@unimi.it (M.P.); 4 Microbiology and Virology Unit, A. Manzoni Hospital, 23900 Lecco, Italy; el.meroni@asst-lecco.it (E.M.); f.luzzaro@asst-lecco.it (F.L.); 5 Biomedical Center, Faculty of Medicine in Pilsen, Charles University, 323 00 Pilsen, Czech Republic; vittoria.mattionimarche01@universitadipavia.it

**Keywords:** KPC-producing, *Escherichia coli*, ST131, WGS, outbreak, H30Rx

## Abstract

KPC-producing *Escherichia coli* (KPC-Ec) remains uncommon, being mainly reported as the cause of sporadic episodes of infection rather than outbreak events. Here we retrospectively describe the dynamics of a large hospital outbreak sustained by KPC-Ec, involving 106 patients and 25 hospital wards, during a six-month period. Twenty-nine representative KPC-Ec isolates (8/29 from rectal swabs; 21/29 from other clinical specimens) have been investigated by Whole-Genome Sequencing (WGS). Outbreak isolates showed a multidrug-resistant profile and harbored several resistance determinants, including *bla*_CTX-M-27_, *aad*A5, *dfr*A17, *sul*I, *gyr*A1AB and *par*C1aAB. Phylogenomic analysis identified the ST131 cluster 1 (23/29 isolates), H30Rx clade C, as responsible for the epidemic event. A further two KPC-Ec ST131 clusters were identified: cluster 2 (*n* = 2/29) and cluster 3 (*n* = 1/29). The remaining KPC-Ec resulted in ST978 (*n* = 2/29) and ST1193 (*n* = 1/29), and were *bla*_KPC-3_ associated. The KPC-Ec ST131 cluster 1, originated in a previous KPC-Kp endemic context probably by plasmid transfer, and showed a clonal dissemination strategy. Transmission of the *bla*_KPC_ gene to the globally disseminated high-risk ST131 clone represents a serious cause of concern. Application of WGS in outbreak investigations could be useful to better understand the evolution of epidemic events in order to address infection control and contrast interventions, especially when high-risk epidemic clones are involved.

## 1. Introduction

The ongoing rise of carbapenemase-producing *Enterobacterales* (CPE) represents an important threat to public health worldwide, in both healthcare and community settings. The pervasive dissemination of CPE substantially impacts on patient safety since few therapeutic alternatives remain. Overall, the worldwide predominant carbapenemase is the Ambler class A *Klebsiella pneumoniae* carbapenemase (KPC) enzyme, encoded by alleles of the *bla*_KPC_ gene, with KPC-producing *K. pneumoniae* (KPC-Kp) being the most common among KPC-producing CPE. Although less prevalent than in *K. pneumoniae*, KPC production in other species belonging to *Enterobacterales* is increasingly reported [1]. The presence of this highly transferable carbapenemase in *Escherichia coli* is of particular concern, being the most common community- and hospital-acquired pathogen, that can be transmitted among humans, animals and in the environment [2]. Moreover, the recent detection of the *bla*_KPC_ gene in *E. coli* belonging to sequence type (ST) 131, a globally disseminated successful clone, represents a cause of serious concern [3]. In fact, the ST131 clone emerged as the most common extraintestinal pathogen that, in association with fluoroquinolone and extended-spectrum cephalosporin resistance, was responsible for the worldwide spread of the extended-spectrum beta-lactamase (ESBL) *bla*_CTX-M-15_ gene. Due to its ability to asymptomatically colonize the gastrointestinal tract of both community and healthcare-associated infections, a stable association of ST131 lineage with *bla*_KPC_ could have relevant consequences for the management of *E. coli* infections [4].

KPC-producing *E. coli* (KPC-Ec) isolates were mainly reported in countries showing a high prevalence of KPC-Kp, probably reflecting a spill-over of resistance genes from the *K. pneumoniae* reservoir [5]. However, acquired carbapenem resistance in *E. coli* is still considered a rare and recent event. The first cases of KPC-Ec were observed in 2004–2005 in the USA (Cleveland, *n* = 1; New York City, *n* = 2; New Jersey, *n* = 1), and in Israel (Tel Aviv, *n* = 4) [3]. After the first detection in Europe—reported in 2008 in France from a patient initially hospitalized in Israel [6]—KPC-Ec has been sporadically reported in the USA [7], Israel [8] and some European countries [9,10].

In Italy, KPC-Kp has been endemic since 2013, but the presence of KPC-Ec isolates remains limited [11]. Results of the most recent Italian Nationwide survey highlighted that KPC was the most frequent carbapenemase from bloodstream infections, mainly in *K. pneumoniae* (95.2%). A recent epidemiological study showed that KPC-Ec were only 1.3% of KPC-producing invasive isolates, but they accounted for 81.4% of cabapenem-resistant *E. coli*, thus highlighting the propensity of these strains to cause invasive infections [12].

Outbreaks caused by KPC-Ec have been described in the USA, Greece, Canada, Israel, Italy and the UK [2,13,14,15,16,17]. Despite this, KPC-Ec has been more frequently reported in sporadic cases than as the cause of outbreaks [18]. In fact, unlike *K. pneumoniae*, in which the KPC determinant is often associated to predominant plasmids and epidemic clones (e.g., IncFII, -FIA, -I2 and ST258), the sporadic appearance of KPC-producing *E. coli* belonging to different genetic backgrounds is largely due to horizontal transfer of different *bla*_KPC_-harboring plasmid groups, thus suggesting a “less successful” association enzyme-pathogen than in KPC-Kp [19].

It is of particular concern that ST131 KPC-Ec strains able to cause outbreaks are emerging, as suggested by the recent reports from Israel, Italy and UK [13,14].

The aim of our study was to report an epidemiological and genomic (Whole-Genome Sequencing, WGS) investigation of a large nosocomial outbreak caused by KPC-Ec, which occurred in Northern Italy in 2016.

## 2. Results

### 2.1. Bacterial Isolates and Phenotypic Characterization

From February to July 2016, 123 KPC-*Escherichia coli* (KPC-Ec) isolates were collected from 106 patients. Twenty-five hospital wards were involved in the outbreak, with those of Internal Medicine (*n* = 34 patients), Cardiology Rehabilitation (*n* = 17), Cardiology (*n* = 10) and Nephrology (*n* = 9) being the most represented. From August 2016 to June 2017, eight additional KPC-Ec were isolated from rectal swabs (*n* = 7) and respiratory secretions (*n* = 1) of seven patients. Four of the above isolates were included in the WGS analysis for comparison. Twenty out of 106 patients belonging to the outbreak period were previously colonized by KPC-*Klebsiella pneumoniae* (KPC-Kp), while in 23 cases the detection of both species was associated, indicating a co-presence at the intestinal level. KPC-Ec was isolated prior to KPC-Kp in seven patients. Six KPC-Kp included in the study were isolated from different wards. Among them, four were from rectal swabs whereas the remaining two were from urine samples. According to both the MicroScan autoSCAN-4 semi-automated system and Sensititre broth microdilution results, representative isolates were consistently resistant to amoxicillin/clavulanate, piperacillin/tazobactam, cefotaxime, ceftazidime, aztreonam, ertapenem, and ceftolozane/tazobactam, but susceptible to colistin, tigecycline and ceftazidime-avibactam. Detailed data concerning the susceptibility results of the 29 KPC-Ec representative isolates are reported in Table 1. Of note, MIC values for imipenem ranged from 4 to >8 mg/L, whereas MICs of meropenem ranged from 4 to 8 mg/L.

### 2.2. Epidemiological Context

Epidemiological surveillance data showed that the epidemic event followed a previous KPC-Kp outbreak, starting in October 2015 and partially overlapping the KPC-Ec outbreak (Supplementary Figure S1). The prevalence of KPC-Ec increased from 0% in 2014 and 2015 to 2.8% in 2016. This trend seems to be related to the increase of KPC-Kp prevalence, from 12.4% in 2014, to 17.3% in 2015 and to 23.1% in 2016 (Supplementary Figure S1). Notably, 50 (47.2%) out of 106 patients’ results were positive to both KPC-Ec and KPC-Kp during previous hospitalizations. In particular, 20 out of 50 patients (40%) were previously colonized by KPC-Kp, while in 23 cases (46%) the isolation of both species was concomitant, indicating the co-presence at the intestinal level. Only in seven patients (14%), however, was KPC-Ec isolated previously with respect to KPC-Kp.

### 2.3. Whole-Genome Sequencing Characterization

Twenty-nine KPC-Ec isolates were further investigated by whole-genome sequencing. They were from urine (*n* = 11), rectal swabs (*n* = 8), blood (*n* = 2), purulent exudate (*n* = 2), respiratory secretions (*n* = 3), drainage fluid (*n* = 1), peritoneal fluid (*n* = 1) and a surgical wound swab (*n* = 1) (Table 2).

Twenty-six out of 29 isolates belonged to ST131 while the remaining three isolates were of ST978 (*n* = 2) and ST1193 (*n* = 1). ST131 isolates carried the following resistance determinants: *aadA5*, *aph(6)-Id, aph(3’’)-Ib, bla*_CTX-M-type_, *bla*_KPC-type_, *bla*_OXA-9_, *bla*_TEM-1A_, *mph(A), sul1, sul2, tet(A)* and *dfrA17* (Table 3). According to the observed quinolone resistance phenotype (see Table 3), ST131 isolates harbored the *parC*1aAB and *gyrA*1AB gene variants. Twenty-four ST131 strains harbored the *bla*_KPC-2_ gene, while only two strains’ results were *bla*_KPC-3_ positive. As shown in Table 3, all but one ST131 strain harbored the *bla*_CTX-M-27_, whereas the remaining one was positive for *bla*_CTX-M-15_. All the ST131 strains showed the O25b:H4 serotype and the fimbrial variant *fimH*30. Both the isolates belonging to ST978 carried the *bla*_KPC-3_ determinant, and showed the serotype O83:H27, and the *fimH*2 variant. The ST1193 isolate harbored the *bla*_KPC-3_ gene, and showed the serotype O75:H5, and the *fimH64* variant.

The virulence genes *sat* (secret autotransporter toxin)*, iss* (increased serum survival) and *gad* (glutamate decarboxylase) were found in all ST131 strains, while the *iha* (adherence protein) determinant was found in all isolates but one ST131. The *senB* (enterotoxin) gene was present in the majority of ST131 isolates, while the *cnf1* (cytotoxic necrotizing factor type 1) was only identified in the isolate sk35y35t, which was the first KPC-Ec that emerged in the outbreak period. A different pattern of virulence genes was detected in the two ST978 strains, consisting of *vat* (vacuolating autotransporter toxin)*, pic* (serine protease) and *gad* determinants. Lastly, the ST1193 isolate harbored *iha*, *sat* and *vat* as virulence genes (Table 3).

**Figure 1:**
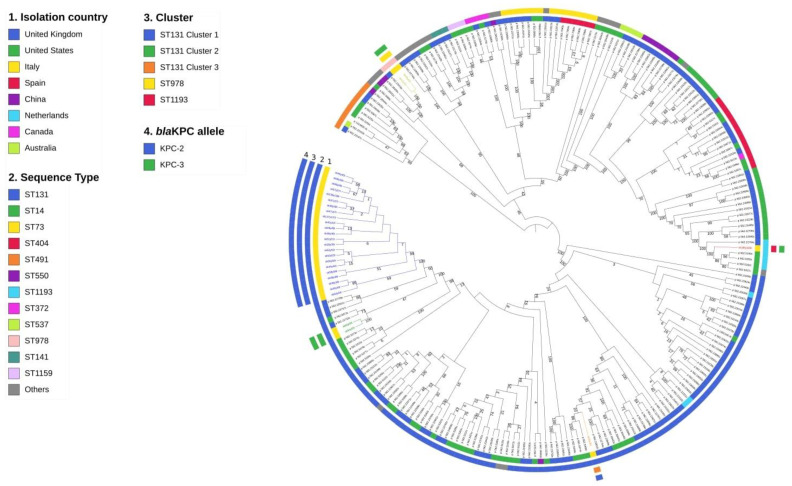
Maximum likelihood phylogenetic tree including the 29 KPC-producing *Escherichia coli* isolates and background strains retrieved from the PATRIC database. The clusters were identified as monophyletic highly supported groups and are reported on the colored inner ring. The geographic origin of all the strains is reported on the second level colored ring. The Sequence Type of the strains (Achtman scheme) is reported on the third colored ring, while the KPC variant is on the outer ring.

The comparison of the 29 KPC-Ec isolates with other *E. coli* genomes deposited in the PATRIC database is shown in Figure 1. Notably, most of them showed a major similarity with KPC-Ec strains collected in the UK. 

**Figure 2:**
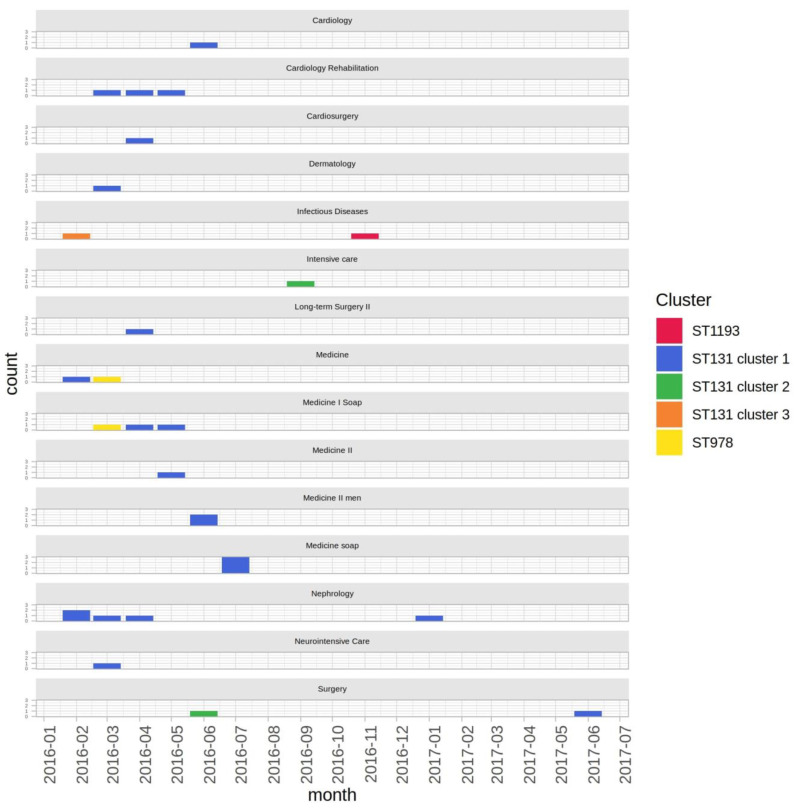
Barplot of the number of isolates per month per ward. Bars were colored on the basis of the phylogenetic clusters shown in Figure 1.

The phylogenetic reconstruction showed the presence of five distinct clusters, three of which belonged to ST131 (“ST131 cluster 1”, “ST131 cluster 2” and “ST131 cluster 3”), one to ST978 and another to ST1193 (Figure 1). The ST131 cluster 1, characteristic of the majority of isolates (*n* = 26), was found in all wards with the exception of the Intensive Care and the Infectious Diseases Units (Figure 2), thus representing the outbreak epidemic clone.

Notably, sporadic isolates belonging to the ST131 cluster 1 were observed till June 2017. Based on phylogenetic data, the first isolate belonging to the ST131 cluster 1 (i.e., the outbreak index strain, coded sk36y36t) was isolated in the Nephrology Ward. The patient had been previously hospitalized for long periods in the past years in both Nephrology and ICU. Of note, the index patient was previously colonized by KPC-Kp (strain code, sk138y138t) (Table 2). The ST131 cluster 2 included two isolates, collected in Surgery and ICU in June and September 2016, respectively. The ST131 cluster 3 was represented by a single isolate, obtained in February 2016 from the Infectious Diseases Ward. ST978 strains were recovered in March 2016 from the blood culture and rectal swab of the same patient (Table 2 and Figure 2). Lastly, the ST1193 strain was isolated in November 2016 in the Infectious Diseases Ward. Figure 2 shows the ward distribution of the KPC-Ec strains belonging to different clusters and STs.

**Figure 3:**
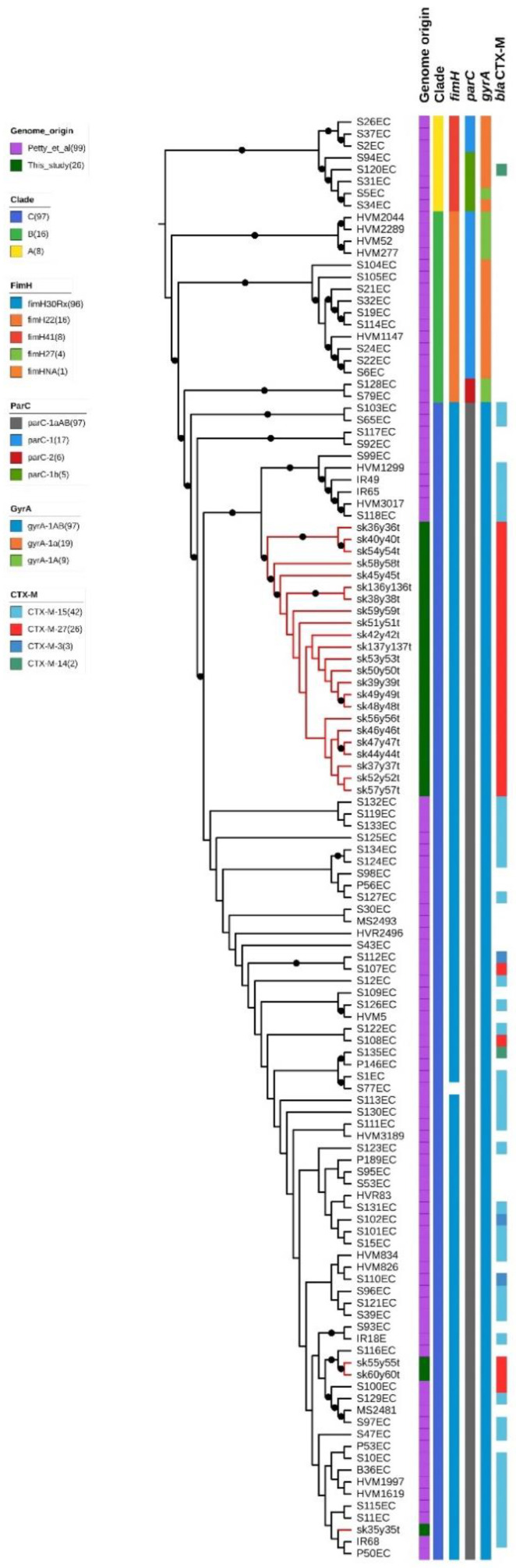
Maximum likelihood phylogenetic tree including the here studied ST131 strains and the ones from Petty et al. [20]. The branches corresponding to the strains of this study are highlighted in red. Metadata have been represented as follows: the first column indicates the origin of the strains; the second one the ST131 clades as called by Petty et al. [20]. The subsequent four columns report the allele variants of the *fimH*, *parC*, *gyrA* and *bla*_CTX-M_ genes.

Recent studies have focused on deciphering the genomic evolution and diversity within the ST131 lineage [4,20,21]. Since three different ST131 clusters were identified, we compared their genomes with those published by Petty and colleagues [20]. As shown in Figure 3, all the studied strains could be assigned to the ST131 clade C, characterized by the fimbrial variant *fimH*30Rx, and the *gyrA*1AB and *parC*1aAB alleles, associated with fluoroquinolone resistance. Phylogeny highlighted that the only difference was the presence of the *bla*_CTX-M-27_ gene variant instead of *bla*_CTX-M-15_ for ST131 clusters 1 and 3.

The six KPC-Kp strains, isolated during the early stages of the outbreak, were investigated for ST and plasmid incompatibility group arrangement. KPC-Kp strains belonged to ST35 (*n* = 2, from Nephrology and Urology), ST17 (*n* = 2, from the Rehabilitation Unit and Internal Medicine), ST3033 (*n* = 1, from Cardiology) and ST2279 (*n* = 1, from Infectious Diseases).

The positive colonization date (and co-isolation date, when it occurred) and related clinical samples of KPC-Ec and KPC-Kp isolates involved in WGS analyses are shown in Supplemental Tables S1 and S2.

**Figure 4:**
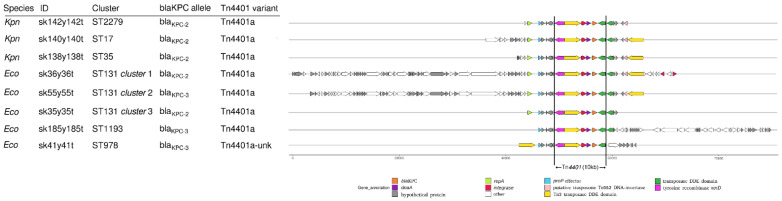
Arrow plot of the different *bla*_KPC_ gene contigs aligned on the Tn4401 transposon (shown inside the vertical lines). The plot includes one representative strain for each *Escherichia coli* cluster and one for each *Klebsiella pneumoniae* ST.

Regarding plasmids, IncFIBpQil was found in all the ST131 and ST1193 KPC-Ec strains, as well as in all the KPC-Kp strains. IncFII was found in all the ST131 cluster 1 and cluster 2 KPC-Ec strains; Col156 was found in all the ST131 cluster 1 isolates but one. IncX3 was detected in both the ST978 isolates. Lastly, all the KPC-Kp strains harbored IncFIB(K)_Kpn3. The KPC-Ec strains belonging to the epidemic ST131 of both cluster 1 and cluster 3 produced the KPC-2 enzyme, as well as the KPC-Kp isolates. The *bla*_KPC-3_ allele was instead found in the ST131 cluster 2, ST1193 and ST978 strains. Analysis of the *bla*_KPC_ surrounding genetic environment showed that all clusters, except ST978 and ST1193, were characterized by a conserved *bla*_KPC_ scaffold (Figure 4). ST978 and ST1193 showed two different scaffolds. All the clusters harbored the Tn4401a, a structural variant of the Tn4401 transposon.

Given the presence of the IncFIBpQil, a well-known KPC-harboring plasmid, the detection of the KPC-2 enzyme, and evaluating the extreme similarity of the blaKPC genomic environment in KPC-Kp and KPC-Ec isolates belonging to the ST131 cluster 1; a plasmid-mediated transmission of the KPC-2 determinant from KPC-Kp to KPC-Ec isolates could reasonably have happened.

Although different temperature conditions were tested, conjugation assay results were negative, thus indicating that the resistance determinants were not located on a conjugative plasmid.

## 3. Discussion 

Our study describes a large intrahospital outbreak caused by KPC-Ec, involving a total of 106 inpatients in 25 wards. To the best of our knowledge, this is the largest outbreak caused by KPC-Ec reported worldwide. Genomic analyses allowed us to ascertain that five different KPC-Ec clusters were involved, three of which belonged to the high-risk ST131 clone. Notably, only the “cluster 1” was responsible for the epidemic event and was associated to the H30Rx clade C sub-clone. The ST131 cluster 1 strains co-harbored *bla*_CTX-M-27_ and *bla*_KPC_ determinants and were the only ones provided with the *senB* virulence gene, coding for the secreted enterotoxin TieB.

Although its relevant ability to spread among patients in different wards, ST131 KPC-Ec showed a low propensity to cause infections. In fact, most samples were collected from rectal swabs, whereas only 20 isolates (16.2%) were obtained from other biological specimens. Of note, only two isolates (1.6%) were from blood cultures. Overall, no deaths were attributable to infection caused by the ST131 KPC-Ec cluster 1 outbreak clone. Conversely, the treatment of infections caused by ST131 KPC-Ec was a challenge. In fact, isolates were mostly nonsusceptible to beta-lactams, including carbapenems and ceftolozane/tazobactam, due to the presence of the *bla*KPC and *bla*CTX-M determinants. KPC-Ec were also mostly resistant to ciprofloxacin and trimethoprim-sulphamethoxazole, another typical feature related to the pandemic ST131 lineage. Therapeutic options were aminoglycosides, tigecycline, colistin and the ceftazidime/avibactam combination. 

The dissemination of the *bla*_KPC_ determinant in *E. coli*, largely due to horizontal transfer of plasmids or other mobile elements into diverse genetic backgrounds, has been previously described [19]. Since the acquisition of *bla*_KPC_ by *E. coli* is a very uncommon event, no data are to date available in the literature about a different prevalence between *bla*_KPC-2_ and *bla*_KPC-3_ variants. Nonetheless, in our experience, the *bla*_KPC-2_ gene seems to be the most represented in Italy, mainly associated with ST131 (unpublished data from a multicentric clinical study). The *bla*_KPC_ genetic background was conserved among both the KPC-Ec ST131 cluster 1 and the KPC-Kp strains of the same period, suggesting that transmission events of plasmid/mobile elements occurred. More importantly, the index patient was colonized during the same time period by both KPC-Kp (strain code sk138y138t) and KPC-Ec (strain code sk36y36t) strains (Table 2), the last one reasonably representing the origin of the outbreak. On the other hand, conjugation assays showed that the *bla*_KPC_ gene was not located on a conjugative plasmid, highlighting that the outbreak was caused by the spread of a dominant clone that had acquired the plasmid, rather than by the dissemination of a resistance plasmid to unrelated strains. Transmissions were drastically interrupted in May 2016, thanks to the adoption of a strict cohorting of both colonized and infected patients, that was assisted by dedicated healthcare staff. During the outbreak period, infection prevention and control measures were implemented. Training courses for the staff based on infection control, contact precautions and hand washing campaigns were promoted. Only sporadic cases related to KPC-Ec (ST131 cluster 1) were observed till June 2017, representing the tail of the outbreak. After this period, no other cases related to KPC-Ec were observed. 

Taking together epidemiological and WGS data, we can speculate that the KPC-Ec outbreak clone developed in the previous context of the KPC-Kp outbreak. Furthermore, the outbreak appeared to be caused by the diffusion of a dominant clone (that probably acquired a *bla*_KPC_-harboring plasmid), and not by the dissemination of a resistance plasmid among the strains of the different clusters. 

Rapid application of WGS in outbreak investigations could be useful to better understand the dynamics of epidemic events in order to address infection control and contrast interventions.

## 4. Materials and Methods

### 4.1. Epidemiological Context and Characterization of Bacterial Isolates 

We retrospectively studied an outbreak caused by KPC-Ec that occurred at the Hospital of Lecco (Northern Italy, close to Milan) across a six-month period (February to July 2016). The hospital accounts for about one thousand beds, and has a catchment area of about 340,000 inhabitants. During the outbreak period, as a part of the surveillance activity of the hospital team for infection control, 123 KPC-Ec nonrepetitive isolates were collected from 106 patients. A total of 103 isolates were from colonization surveillance rectal swabs. Rectal swabs have been used to screen intestinal colonization by KPC-Ec, as indicated by international guidelines (Centers for Diseases Control and Prevention, CDC, 2015; https://www.cdc.gov/hai/pdfs/cre/cre-guidance-508.pdf, accessed on 14 June 2021). Twenty strains from other sites were isolated from symptomatic patients with suspected infection. These isolates were from urine (*n* = 11), blood (*n* = 2), purulent exudate (*n* = 2), respiratory secretions (*n* = 2), drainage fluid (*n* = 1), peritoneal fluid (*n* = 1) and a surgical wound swab (*n* = 1). Strains from the same patients were included only when isolated from different sites. A further four KPC-Ec isolates were sporadically collected after the epidemic event and until June 2017 and were also investigated to verify their clonal relationship to outbreak strains. To better clarify the origin of the *bla*_KPC_ gene in the KPC-Ec strains, six KPC-Kp isolates were collected from patients cocolonized by KPC-Kp and KPC-Ec and were included in the study. Of them, two were obtained at the beginning of the episode (including those isolated from the index patient), while the remaining were collected during the outbreak from patients admitted to those wards that were mainly involved in the episode. Isolates from rectal swabs were screened for carbapenemase production using chromogenic Brilliance CRE agar (Thermo Fisher Scientific). Bacterial isolates were identified to the species level using MALDI-TOF mass spectrometry (Vitek MS, bioMérieux), while susceptibility testing was routinely determined by the Vitek 2 system (bioMérieux). Isolates suspected of carbapenemase production (MIC values for ertapenem and/or meropenem >0.125 mg/L) were evaluated to assess the presence of specific carbapenem resistance determinants using the immunochromatographic technique (RESIST-4 O.K.N.V., Coris BioConcept) and/or a molecular dedicated assay (Xpert Carba-R, Cepheid). 

To characterize epidemic isolates and better understand the dynamics of the outbreak, a total of 29 KPC-Ec strains (four of which were sporadically isolated in the *post*-outbreak period) were selected as representatives based on the site of infection or colonization, date and ward of admission (Table 2). MIC values of these isolates were determined by the MicroScan autoSCAN-4 system (NMDRM1 panel, Beckman Coulter). Selected antimicrobials (i.e., ceftazidime-avibactam, ceftolozane/tazobactam, and colistin) were evaluated by a broth microdilution Sensititre panel used for multidrug-resistant Gram-negative strains (DKMGN panel, Thermo Fisher Scientific). EUCAST criteria were used for determining susceptibility categories [22]. Finally, these isolates were analyzed by WGS-based typing and SNP-based phylogenetic reconstruction.

### 4.2. High Resolution Melting Assay

The selection of KPC-producing *K. pneumoniae* strains chosen for WGS investigation was made on the basis of the High Resolution Melting (HRM) assay results. The HRM was performed on the *wzi* hypervariable capsular gene as described by Perini et al. [23] using MeltingPlot software [24].

### 4.3. Whole-Genome Sequencing

A total of 35 strains (29 KPC-Ec and 6 KPC-Kp), representative of the epidemic event and of the post outbreak period, were processed for WGS analysis. In detail, inclusion criteria were: (i) isolates from ascertained infections (other than from screening rectal swabs) were chosen preferentially; (ii) KPC-Ec from all hospital wards involved in the epidemic event; (iii) KPC-Ec isolated in different periods of the outbreak (at the beginning, medium period, tail of the outbreak); (iv) In addition, 8 KPC-Ec from rectal swabs were included in order to evaluate the presence of the outbreak clone in the patients’ intestinal microbiota. Genomic DNA was extracted using a QIAamp DNA minikit (Qiagen) following the manufacturer’s instructions and sequenced using the Illumina Miseq platform with a 2 × 250 paired-end run after Nextera XT library preparation (Illumina Inc., San Diego, CA, USA). 

### 4.4. CoreSNP Calling and Phylogenetic Analyses

For each of the 35 strains included in the study, the reads quality was assessed using FastqC software (https://www.bioinformatics.babraham.ac.uk/projects/fastqc/, accessed on 29 June 2020), and the low quality terminal bases were trimmed using Trimmomatic software [25]. Reads were assembled using SPAdes software [26].

All the genome assemblies were submitted to the European Nucleotide Archive (ENA) with the project code PRJEB40388. All the ID codes are listed in Table S3.

The genome distance of Each KPC-Ec genome assembly was estimated using Mash software [27] against a collection of 3325 *E. coli* genomes retrieved from the PATRIC database [28] and the 50 most similar genomes were selected for subsequent analyses. All the selected genome assemblies (from this study and the PATRIC database) were aligned against the *E. coli* MG1655 reference genome using progressive Mauve and coreSNPs were called as described by Gona and colleagues [29]. Repeated regions in the reference genome assembly were detected using Blastn. Then coreSNPs localized within repeated regions were masked. From here, the obtained coreSNP alignment was called “Global coreSNP”.

Global coreSNP alignments were subjected to phylogenetic analysis using RAxML software with a 100 pseudo-bootstrap, after best model selection using ModelTest-NG [30]. The strains were then clustered on the basis of the Global phylogenetic tree and SNP distance. At first, we identified on the ML phylogenetic tree the largest highly supported (>75 bootstrap) monophyletic groups including study strains only. 

### 4.5. Whole-Genome Sequencing-Based Typing

Resistance genes of the 29 KPC-Ec strains were identified using the ResFinder online tool [31] and SRST2 software [32] with the ARG-ANNOT dataset [33]. Virulence genes were detected by the VirulenceFinder online tool [34]. Plasmid incompatibility groups were detected using PlasmidFinder [35]. The Multi Locus Sequence Typing profiles of the 29 *E. coli* and six *K. pneumoniae* strains were determined in silico according to the Achtman and Pasteur schemes, respectively, using an in-house Perl script.

### 4.6. KPC-Harboring Contigs Comparison

For each identified cluster (see above) one representative strain was selected and the genome assembly was analyzed as follows. The contig harboring *bla*_KPC_ gene was identified by Blastn search (E-value threshold: 0.00001). The extracted contigs were oriented on the basis of *bla*_KPC_ gene orientation, then annotated using Prokka [36] and aligned with progressive Mauve [37]. The Tn4401 transposon was annotated using TETyper [38]. Lastly, the gene composition and synteny of extracted contigs were graphically represented using the R library genoPlotR [39].

### 4.7. Conjugation Assay

To assess the possible transferability of resistance determinants identified, a conjugation assay was performed using the *E. coli* J53 Azide^R^ as the recipient strain at temperatures of both 25 °C and 37 °C, and with MER 0.5mg/L for the selection of transconjugants.

## 5. Conclusions

Although KPC-producing *Escherichia coli* (KPC-Ec) remains uncommon, and mainly reported as the cause of sporadic episodes of infection rather than outbreaks, the present work shows that the acquisition of *bla*_KPC_ gene by a high-risk successful clone, as the ST131, can lead to even large and potentially difficult to manage epidemic events. The attention on the presence and circulation of carbapenemase-producing enterobacteria (CPE) should be always kept high, especially in healthcare settings. In this context, the application of WGS could be useful to better understand the evolution and dynamic of outbreaks sustained by CPE in order to promptly address infection control and contrast interventions.

## Figures and Tables

**Table 1:** Antimicrobial susceptibility profiles of selected KPC-positive *E. coli* strains.

ID Strain	Antimicrobial Susceptibility (MIC, µg/mL)																
	AMC	PTZ	CTX	CAZ	CZA	C/T	MER	IMI	ERT	AMK	GNT	AZT	CIP	TBR	TIG	SXT	COL
sk46y46t	>32 (R)	>16 (R)	>32 (R)	16 (R)	<0,5/4 (S)	4/4 (R)	4 (I)	8 (I)	>1 (R)	<8 (S)	≤2 (S)	>4 (R)	>1 (R)	≤2 (S)	≤1 (S)	>4/76 (R)	<0,25 (S)
**sk36y36t**	>32 (R)	>16 (R)	>32 (R)	32 (R)	<0,5/4 (S)	4/4 (R)	8 (I)	8 (I)	>1 (R)	<8 (S)	≤2 (S)	>4 (R)	>1 (R)	4 (I)	≤1 (S)	>4/76 (R)	<0,25 (S)
sk35y35t	>32 (R)	>16 (R)	>32 (R)	>32 (R)	<0,5/4 (S)	4/4 (R)	8 (I)	8 (I)	>1 (R)	<8 (S)	>4 (R)	>4 (R)	>1 (R)	>4 (R)	≤1 (S)	≤2/38 (S)	0,5 (S)
sk39y39t	>32 (R)	>16 (R)	>32 (R)	>32 (R)	<0,5/4 (S)	2/4 (R)	8 (I)	8 (I)	>1 (R)	<8 (S)	≤2 (S)	>4 (R)	>1 (R)	≤2 (S)	≤1 (S)	>4/76 (R)	0,5 (S)
sk37y37t	>32 (R)	>16 (R)	>32 (R)	8 (R)	<0,5/4 (S)	2/4 (R)	4 (I)	4 (I)	>1 (R)	<8 (S)	≤2 (S)	>4 (R)	>1 (R)	≤2 (S)	≤1 (S)	>4/76 (R)	0,5 (S)
sk38y38t	>32 (R)	>16 (R)	>32 (R)	>32 (R)	<0,5/4 (S)	2/4 (R)	8 (I)	8 (I)	>1 (R)	<8 (S)	≤2 (S)	>4 (R)	>1 (R)	≤2 (S)	≤1 (S)	>4/76 (R)	0,5 (S)
sk42y42t	>32 (R)	>16 (R)	>32 (R)	>32 (R)	<0,5/4 (S)	4/4 (R)	8 (I)	8 (I)	>1 (R)	16 (I)	≤2 (S)	>4 (R)	>1 (R)	4 (I)	≤1 (S)	>4/76 (R)	0,5 (S)
sk40y40t	>32 (R)	>16 (R)	>32 (R)	32 (R)	<0,5/4 (S)	4/4 (R)	8 (I)	8 (I)	>1 (R)	<8 (S)	4 (I)	>4 (R)	>1 (R)	≤2 (S)	≤1 (S)	>4/76 (R)	0,5 (S)
sk47y47t	>32 (R)	>16 (R)	>32 (R)	>32 (R)	<0,5/4 (S)	4/4 (R)	8 (I)	>8 (R)	>1 (R)	16 (I)	≤2 (S)	>4 (R)	>1 (R)	4 (I)	≤1 (S)	>4/76 (R)	0,5 (S)
sk41y41t	>32 (R)	>16 (R)	>32 (R)	>32 (R)	<0,5/4 (S)	16/4 (R)	8 (I)	8 (I)	>1 (R)	>16 (R)	4 (I)	>4 (R)	≤0,06 (S)	>4 (R)	≤1 (S)	≤2/38 (S)	<0,25 (S)
sk43y43t	>32 (R)	>16 (R)	>32 (R)	>32 (R)	<0,5/4 (S)	32/4 (R)	8 (I)	8 (I)	>1 (R)	>16 (R)	4 (I)	>4 (R)	≤0,06 (S)	>4 (R)	≤1 (S)	≤2/38 (S)	0,5 (S)
sk54y54t	>32 (R)	>16 (R)	>32 (R)	32 (R)	<0,5/4 (S)	4/4 (R)	8 (I)	>8 (R)	>1 (R)	<8 (S)	≤2 (S)	>4 (R)	>1 (R)	4 (I)	≤1 (S)	≤2/38 (S)	0,5 (S)
sk44y44t	>32 (R)	>16 (R)	>32 (R)	16 (R)	<0,5/4 (S)	4/4 (R)	8 (I)	8 (I)	>1 (R)	<8 (S)	≤2 (S)	>4 (R)	>1 (R)	≤2 (S)	≤1 (S)	>4/76 (R)	0,5 (S)
sk45y45t	>32 (R)	>16 (R)	>32 (R)	>32 (R)	<0,5/4 (S)	2/4 (R)	8 (I)	8 (I)	>1 (R)	<8 (S)	≤2 (S)	>4 (R)	>1 (R)	≤2 (S)	≤1 (S)	>4/76 (R)	0,5 (S)
sk48y48t	>32 (R)	>16 (R)	>32 (R)	16 (R)	<0,5/4 (S)	2/4 (R)	8 (I)	8 (I)	>1 (R)	<8 (S)	4 (I)	>4 (R)	>1 (R)	≤2 (S)	≤1 (S)	>4/76 (R)	<0,25 (S)
sk49y49t	>32 (R)	>16 (R)	>32 (R)	>32 (R)	<0,5/4 (S)	2/4 (R)	8 (I)	>8 (R)	>1 (R)	<8 (S)	≤2 (S)	>4 (R)	>1 (R)	≤2 (S)	≤1 (S)	≤2/38 (S)	<0,25 (S)
sk51y51t	>32 (R)	>16 (R)	>32 (R)	>32 (R)	<0,5/4 (S)	2/4 (R)	8 (I)	8 (I)	>1 (R)	<8 (S)	≤2 (S)	>4 (R)	>1 (R)	≤2 (S)	≤1 (S)	>4/76 (R)	<0,25 (S)
sk50y50t	>32 (R)	>16 (R)	>32 (R)	16 (R)	<0,5/4 (S)	4/4 (R)	8 (I)	4 (I)	>1 (R)	<8 (S)	≤2 (S)	>4 (R)	>1 (R)	≤2 (S)	≤1 (S)	>4/76 (R)	0,5 (S)
sk52y52t	>32 (R)	>16 (R)	>32 (R)	16 (R)	<0,5/4 (S)	2/4 (R)	8 (I)	8 (I)	>1 (R)	<8 (S)	4 (I)	>4 (R)	>1 (R)	4 (I)	≤1 (S)	>4/76 (R)	<0,25 (S)
sk53y53t	>32 (R)	>16 (R)	>32 (R)	32 (R)	<0,5/4 (S)	4/4 (R)	8 (I)	8 (I)	>1 (R)	<8 (S)	≤2 (S)	>4 (R)	>1 (R)	≤2 (S)	≤1 (S)	>4/76 (R)	0,5 (S)
sk56y56t	>32 (R)	>16 (R)	>32 (R)	16 (R)	<0,5/4 (S)	2/4 (R)	8 (I)	8 (I)	>1 (R)	<8 (S)	≤2 (S)	>4 (R)	>1 (R)	≤2 (S)	≤1 (S)	>4/76 (R)	0,5 (S)
sk55y55t	>32 (R)	>16 (R)	>32 (R)	>32 (R)	<0,5/4 (S)	16/4 (R)	4 (I)	4 (I)	>1 (R)	<8 (S)	≤2 (S)	>4 (R)	>1 (R)	≤2 (S)	≤1 (S)	≤2/38 (S)	0,5 (S)
sk57y57t	>32 (R)	>16 (R)	>32 (R)	8 (R)	<0,5/4 (S)	4/4 (R)	8 (I)	8 (I)	>1 (R)	<8 (S)	≤2 (S)	>4 (R)	>1 (R)	≤2 (S)	≤1 (S)	>4/76 (R)	<0,25 (S)
sk58y58t	>32 (R)	>16 (R)	>32 (R)	8 (R)	<0,5/4 (S)	2/4 (R)	4 (I)	4 (I)	>1 (R)	<8 (S)	≤2 (S)	>4 (R)	>1 (R)	≤2 (S)	≤1 (S)	>4/76 (R)	0,5 (S)
sk59y59t	>32 (R)	>16 (R)	>32 (R)	>32 (R)	<0,5/4 (S)	4/4 (R)	8 (I)	8 (I)	>1 (R)	<8 (S)	≤2 (S)	>4 (R)	>1 (R)	≤2 (S)	≤1 (S)	>4/76 (R)	0,5 (S)
sk60y60t	>32 (R)	>16 (R)	>32 (R)	>32 (R)	<0,5/4 (S)	32/4 (R)	8 (I)	8 (I)	>1 (R)	<8 (S)	≤2 (S)	>4 (R)	>1 (R)	≤2 (S)	≤1 (S)	>4/76 (R)	1 (S)
sk185y185t	>32 (R)	>16 (R)	>32 (R)	>32 (R)	<0,5/4 (S)	4/4 (R)	8 (I)	8 (I)	>1 (R)	<8 (S)	≤2 (S)	>4 (R)	>1 (R)	≤2 (S)	≤1 (S)	>4/76 (R)	0,5 (S)
sk136y136t	>32 (R)	>16 (R)	>32 (R)	32 (R)	<0,5/4 (S)	2/4 (R)	8 (I)	8 (I)	>1 (R)	<8 (S)	≤2 (S)	>4 (R)	>1 (R)	≤2 (S)	≤1 (S)	>4/76 (R)	0,5 (S)
sk137y137t	>32 (R)	>16 (R)	>32 (R)	>32 (R)	<0,5/4 (S)	4/4 (R)	8 (I)	8 (I)	>1 (R)	<8 (S)	≤2 (S)	>4 (R)	>1 (R)	≤2 (S)	≤1 (S)	>4/76 (R)	0,5 (S)

MIC: minimum inhibitory concentration; AMC: amoxicillin/clavulanate; PTZ: piperacillin/tazobactam; CTX: cefotaxime; CAZ: ceftazidime; MER: meropenem; IMI: imipenem; ERT: ertapenem; AMK: amikacin; GNT: gentamicin; AZT: aztreonam; CIP: ciprofloxacin; TBR: tobramycin; TIG: tigecycline; SXT: trimethoprim-sulfamethoxazole; CZA: ceftazidime/avibactam; C/T: ceftolozane/tazobactam; COL: colistin; S: susceptible; I: intermediate; R: resistant. Susceptibility results were interpreted according to the European Committee on Antimicrobial Susceptibility Testing (EUCAST, 2016) criteria. The “index strain” is indicated in bold.

**Table 2:** Main metadata of the 35 clinical samples included in the study and subjected to whole-genome sequencing. The “index strain” is indicated in bold.

ID Sample	Microorganism	Ward	Isolation Date	Material
sk35y35t	*Escherichia coli*	Infectious Diseases	15 February 2016	Rectal swab
**sk36y36t**	*Escherichia coli*	Nephrology	16 February 2016	Rectal swab
sk37y37t	*Escherichia coli*	Medicine	26 February 2016	Urine
sk38y38t	*Escherichia coli*	Nephrology	29 February 2016	Rectal swab
sk39y39t	*Escherichia coli*	Cardiology Rehabilitation	1 March 2016	Urine
sk40y40t	*Escherichia coli*	Neurointensive Care	3 March 2016	Purulent exudate
sk42y42t	*Escherichia coli*	Dermatology	3 March 2016	Urine
sk41y41t	*Escherichia coli*	Medicine I Soap	8 March 2016	Blood
sk43y43t	*Escherichia coli*	Medicine	17 March 2016	Rectal swab
sk44y44t	*Escherichia coli*	Nephrology	23 March 2016	Blood
sk45y45t	*Escherichia coli*	Cardiology Rehabilitation	5 April 2016	Urine
sk46y46t	*Escherichia coli*	Cardiosurgery	18 April 2016	Bronchial aspirate
sk47y47t	*Escherichia coli*	Medicine I Soap	18 April 2016	Urine
sk48y48t	*Escherichia coli*	Nephrology	21 April 2016	Surgical wound swab
sk49y49t	*Escherichia coli*	Long-term Surgery II	24 April 2016	Peritoneal fluid
sk50y50t	*Escherichia coli*	Medicine II	10 May 2016	Respiratory secretion
sk51y51t	*Escherichia coli*	Cardiology Rehabilitation	13 May 2016	Urine
sk52y52t	*Escherichia coli*	Medicine I Soap	22 May 2016	Urine
sk53y53t	*Escherichia coli*	Medicine II men	11 June 2016	Urine
sk54y54t	*Escherichia coli*	Medicine II men	25 June 2016	Urine
sk55y55t	*Escherichia coli*	Surgery	29 June 2016	Drainage fluid
sk56y56t	*Escherichia coli*	Cardiology	30 June 2016	Urine
sk57y57t	*Escherichia coli*	Medicine soap	13 July 2016	Urine
sk58y58t	*Escherichia coli*	Medicine soap	13 July 2016	Rectal swab
sk59y59t	*Escherichia coli*	Medicine soap	14 July 2016	Purulent exudate
sk60y60t	*Escherichia coli*	Intensive care	30 September 2016	Rectal swab
sk185y185t	*Escherichia coli*	Infectious Diseases	14 November 2016	Respiratory secretion
sk136y136t	*Escherichia coli*	Nephrology	23 January 2017	Rectal swab
sk137y137t	*Escherichia coli*	Surgery	5 June 2017	Rectal swab
sk138y138t	*Klebsiella pneumoniae*	Nephrology	22 January 2016	Urine
sk139y139t	*Klebsiella pneumoniae*	Cardiology	16 February 2016	Rectal swab
sk140y140t	*Klebsiella pneumoniae*	Rehabilitation Medicine	25 February 2016	Rectal swab
sk141y141t	*Klebsiella pneumoniae*	Urology	25 February 2016	Urine
sk142y142t	*Klebsiella pneumoniae*	Infectious Diseases	22 March 2016	Rectal swab
sk143y143t	*Klebsiella pneumoniae*	Medicine	31 March 2016	Rectal swab

**Table 3:** Genomic characteristics of the sequenced isolates. The “index strain” is indicated in bold.

ID Sample	Isolation Date	MLST	Cluster	Serotype	*fimH*	Resistance Genes	Virulence Genes	Plasmid Incompatibility Groups
sk35y35t	15 February 2016	131	ST131 cluster 3	O25b:H4	30	*ampH, bla*_TEM-90_ *, bla*_KPC-2,_ *blaOXA-9, bla*_CTX-M-15_ *, ampC2, catB4, bla*_OXA-1_ *, aac(3)-IIa, parC1aAB, gyrA1AB*	*iha, sat, iss, cnf1, gad*	IncFIB(pQil)_1_pQil_JN233705
**sk36y36t**	16 February 2016	131	ST131 cluster 1	O25b:H4	30	*strA, ampH, bla*_TEM-122_ *, bla*_KPC-2_ *, sulII, bla*_OXA-9_ *, bla*_CTX-M-27_ *, ampC2, aadA5, mphA, tetR, dfrA17, tetA, sulI, strB, parC1aAB, gyrA1AB*	*iha, sat, iss, senB, gad*	IncFIB(pQil)_1_pQil_JN233705, Col156_1_NC_009781
sk37y37t	26 February 2016	131	ST131 cluster 1	O25b:H4	30	*strA, ampH, bla*_TEM-90_ *, bla*_KPC-2_ *, sulII, bla*_OXA-9_ *, bla*_CTX-M-27_ *, ampC2, aadA5, mphA, tetR, dfrA17, tetA, sulI, strB, parC1aAB, gyrA1AB*	*iha, sat, iss, senB, gad*	IncFIB(pQil)_1_pQil_JN233705, Col156_1_NC_009781
sk38y38t	29 February 2016	131	ST131 cluster 1	O25b:H4	30	*strA, ampH, bla*_TEM-122_ *, bla*_KPC-2_ *, sulII, bla*_OXA-9_ *, bla*_CTX-M-27_ *, ampC2, aadA5, mphA, tetR, dfrA17, tetA, sulI, strB, parC1aAB, gyrA1AB*	*iha, sat, iss, senB, gad*	IncFIB(pQil)_1_pQil_JN233705, Col156_1_NC_009781
sk39y39t	1 March 2016	131	ST131 cluster 1	O25b:H4	30	*strA, ampH, bla*_TEM-141_ *, bla*_KPC-2_ *, sulII, bla*_OXA-9_ *, bla*_CTX-M-27_ *, ampC2, aadA5, mphA, tetR, dfrA17, tetA, sulI, strB, parC1aAB, gyrA1AB*	*iha, sat, iss, senB, gad*	IncFIB(pQil)_1_pQil_JN233705, Col156_1_NC_009781
sk40y40t	3 March 2016	131	ST131 cluster 1	O25b:H4	30	*strA, ampH, bla*_TEM-122_ *, bla*_KPC-2_ *, sulII, bla*_OXA-9_ *, bla*_CTX-M-27_ *, ampC2, aadA5, mphA, tetR, dfrA17, tetA, sulI, strB, parC1aAB, gyrA1AB*	*iha, sat, iss, senB, gad*	IncFIB(pQil)_1_pQil_JN233705, Col156_1_NC_009781
sk42y42t	3 March 2016	131	ST131 cluster 1	O25b:H4	30	*strA, ampH, bla*_TEM-79_ *, bla*_KPC-2_ *, sulII, bla*_OXA-9_ *, bla*_CTX-M-27_ *, ampC2, aadA5, mphA, tetR, dfrA17, tetA, sulI, strB, parC1aAB, gyrA1AB*	*iha, sat, iss, senB, gad*	IncFIB(pQil)_1_pQil_JN233705, Col156_1_NC_009781
sk41y41t	8 March 2016	978	ST978	O83:H27	2	*aac(6)-Ib, ampH, ampC2, bla*_KPC-3_	*vat, pic, gad*	IncX3_1__JN247852
sk43y43t	17 March 2016	978	ST978	O83:H27	2	*aac(6)-Ib, ampH, ampC2, bla*_KPC-3_	*vat, pic, gad*	IncX3_1__JN247852
sk44y44t	23 March 2016	131	ST131 cluster 1	O25b:H4	30	*strA, ampH, bla*_TEM-122_ *, bla*_KPC-2_ *, sulII, bla*_OXA-9_ *, bla*_CTX-M-27_ *, ampC2, aadA5, mphA, tetR, dfrA17, tetA, sulI, strB, parC1aAB, gyrA1AB*	*iha, sat, iss, senB, gad*	IncFIB(pQil)_1_pQil_JN233705, Col156_1_NC_009781
sk45y45t	5 April 2016	131	ST131 cluster 1	O25b:H4	30	*strA, ampH, bla*_TEM-79_ *, bla*_KPC-2_ *, sulII, bla*_OXA-9_ *, bla*_CTX-M-27_ *, ampC2, aadA5, mphA, tetR, dfrA17, tetA, sulI, strB, parC1aAB, gyrA1AB*	*iha, sat, iss, senB, gad*	IncFIB(pQil)_1_pQil_JN233705, Col156_1_NC_009781
sk46y46t	18 April 2016	131	ST131 cluster 1	O25b:H4	30	*strA, ampH, bla*_TEM-156_ *, bla*_KPC-2_ *, sulII, bla*_OXA-9_ *, bla*_CTX-M-27_ *, ampC2, aadA5, mphA, tetR, dfrA17, tetA, sulI, strB, parC1aAB, gyrA1AB*	*iha, sat, iss, senB, gad*	IncFIB(pQil)_1_pQil_JN233705, Col156_1_NC_009781
sk47y47t	18 April 2016	131	ST131 cluster 1	O25b:H4	30	*strA, ampH, bla*_TEM-54_ *, bla*_KPC-2_ *, sulII, bla*_OXA-9_ *, bla*_CTX-M-27_ *, ampC2, aadA5, mphA, tetR, dfrA17, tetA, sulI, strB, parC1aAB, gyrA1AB*	*iha, sat, iss, senB, gad*	IncFIB(pQil)_1_pQil_JN233705, Col156_1_NC_009781
sk48y48t	21 April 2016	131	ST131 cluster 1	O25b:H4	30	*strA, ampH, bla*_TEM-192_ *, bla*_KPC-2_ *, sulII, bla*_OXA-9_ *, bla*_CTX-M-27_ *, ampC2, aadA5, mphA, tetR, dfrA17, tetA, sulI, strB, parC1aAB, gyrA1AB*	*iha, sat, iss, senB, gad*	IncFIB(pQil)_1_pQil_JN233705, Col156_1_NC_009781
sk49y49t	24 April 2016	131	ST131 cluster 1	O25b:H4	30	*ampH, bla*_TEM-122_ *, bla*_KPC-2_ *, bla*_OXA-9_ *, bla*_CTX-M-27_ *, ampC2, parC1aAB, gyrA1AB*	*iha, sat, iss, senB, gad*	IncFIB(pQil)_1_pQil_JN233705, Col156_1_NC_009781
sk50y50t	10 May 2016	131	ST131 cluster 1	O25b:H4	30	*strA, ampH, bla*_TEM-122_ *, bla*_KPC-2_ *, sulII, bla*_OXA-9_ *, bla*_CTX-M-27_ *, ampC2, aadA5, mphA, tetR, dfrA17, tetA, sulI, strB, parC1aAB, gyrA1AB*	*iha, sat, iss, senB, gad*	IncFIB(pQil)_1_pQil_JN233705, Col156_1_NC_009781
sk51y51t	13 May 2016	131	ST131 cluster 1	O25b:H4	30	*strA, ampH, bla*_TEM-79_ *, bla*_KPC-2_ *, sulII, bla*_OXA-9_ *, bla*_CTX-M-27_ *, ampC2, aadA5, mphA, tetR, dfrA17, tetA, sulI, strB, parC1aAB, gyrA1AB*	*iha, sat, iss, senB, gad*	IncFIB(pQil)_1_pQil_JN233705, Col156_1_NC_009781
sk52y52t	22 May 2016	131	ST131 cluster 1	O25b:H4	30	*strA, ampH, bla*_TEM-150_ *, bla*_KPC-2_ *, sulII, bla*_OXA-9_ *, bla*_CTX-M-27_ *, ampC2, aadA5, mphA, tetR, dfrA17, tetA, sulI, strB, parC1aAB, gyrA1AB*	*iha, sat, iss, senB, gad*	IncFIB(pQil)_1_pQil_JN233705, Col156_1_NC_009781
sk53y53t	11 June 2016	131	ST131 cluster 1	O25b:H4	30	*strA, ampH, bla*_TEM-168_ *, bla*_KPC-2_ *, sulII, bla*_OXA-9_ *, bla*_CTX-M-27_ *, ampC2, aadA5, mphA, tetR, dfrA17, tetA, sulI, strB, parC1aAB, gyrA1AB*	*iha, sat, iss, senB, gad*	IncFIB(pQil)_1_pQil_JN233705, Col156_1_NC_009781
sk54y54t	25 June 2016	131	ST131 cluster 1	O25b:H4	30	*ampH, bla*_TEM-122_*, bla*_KPC-2_*, bla*OXA-9*, bla*_CTX-M-27,_ *ampC2, parC1aAB, gyrA1AB*	*iha, sat, iss, senB, gad*	IncFIB(pQil)_1_pQil_JN233705
sk55y55t	29 June 2016	131	ST131 cluster 2	O25b:H4	30	*ampH, bla*_TEM-79_ *, bla*_KPC-3_ *, bla*_OXA-9_ *, bla*_CTX-M-27_ *, ampC2, parC1aAB, gyrA1AB*	*sat, iss, gad*	IncFIB(pQil)_1_pQil_JN233705
sk56y56t	30 June 2016	131	ST131 cluster 1	O25b:H4	30	*strA, ampH, bla*_TEM-79_ *, bla*_KPC-2_ *, sulII, bla*_OXA-9_ *, bla*_CTX-M-27_ *, ampC2, aadA5, mphA, tetR, dfrA17, tetA, sulI, strB, parC1aAB, gyrA1AB*	*iha, sat, iss, senB, gad*	IncFIB(pQil)_1_pQil_JN233705, Col156_1_NC_009781, Col(BS512)_1_NC_010656
sk57y57t	13 July 2016	131	ST131 cluster 1	O25b:H4	30	*strA, ampH, bla*_TEM-168_ *, bla*_KPC-2_ *, sulII, bla*_OXA-9_ *, bla*_CTX-M-27_ *, ampC2, aadA5, mphA, tetR, dfrA17, tetA, sulI, strB, parC1aAB, gyrA1AB*	*iha, sat, iss, senB, gad*	IncFIB(pQil)_1_pQil_JN233705, Col156_1_NC_009781
sk58y58t	13 July 2016	131	ST131 cluster 1	O25b:H4	30	*strA, ampH, bla*_TEM-141_ *, bla*_KPC-2_ *, sulII, bla*_OXA-9_ *, bla*_CTX-M-27_ *, ampC2, aadA5, mphA, tetR, dfrA17, tetA, sulI, strB, parC1aAB, gyrA1AB*	*iha, sat, iss, senB, gad*	IncFIB(pQil)_1_pQil_JN233705, Col156_1_NC_009781
sk59y59t	14 July 2016	131	ST131 cluster 1	O25b:H4	30	*strA, ampH, bla_TEM-79_, bla_KPC-2_, sulII, bla_OXA-9_, bla_CTX-M-27_, ampC2, aadA5, mphA, tetR, dfrA17, tetA, sulI, strB, parC1aAB, gyrA1AB*	*iha, sat, iss, senB, gad*	IncFIB(pQil)_1_pQil_JN233705, Col156_1_NC_009781
sk60y60t	30 September 2016	131	ST131 cluster 2	O25b:H4	30	*ampH, bla*_TEM-79_ *, bla*_KPC-3_ *, bla*_OXA-9_ *, bla*_CTX-M-27_ *, ampC2, parC1aAB, gyrA1AB*	*sat, iss, gad*	IncFIB(pQil)_1_pQil_JN233705
sk185y185t	14 November 2016	1193	ST1193	O75:H5	64	*qnrB1, bla*_TEM-198_ *, bla*_KPC-3_ *, ampC2, aac(3)-IId, dfrA14*	*iha, sat, vat*	IncFIB(pQil)_1_pQil_JN233705, Col(BS512)_1_NC_010656
sk136y136t	23 January 2017	131	ST131 cluster 1	O25b:H4	30	*strA, ampH, bla*_TEM-168_ *, bla*_KPC-2_ *, sulII, bla*_OXA-9_ *, bla*_CTX-M-27_ *, ampC2, aadA5, mphA, tetR, dfrA17, tetA, sulI, strB, parC1aAB, gyrA1AB*	*iha, sat, iss, senB, gad*	IncFIB(pQil)_1_pQil_JN233705, Col156_1_NC_009781
sk137y137t	5 June 2017	131	ST131 cluster 1	O25b:H4	30	*ampH, bla*_TEM-150_*, bla*_KPC-2_*, bla*_OXA-9_*, bla*_CTX-M-27_, *ampC2, parC1aAB, gyrA1AB*	*iha, sat, iss, senB, gad*	IncFIB(pQil)_1_pQil_JN233705, Col156_1_NC_009781
sk138y138t	22 January 2016	35				*oqxBgb, bla*_KPC-2_ *, bla*_TEM-79_ *, bla*_OXA-9_ *, oqxA, tetD, ampH*		IncFIB(pQil)_1_pQil_JN233705, IncFIB(K)_1_Kpn3_JN233704
sk139y139t	16 February 2016	3033				*strA, strB, oqxBgb, catB4, bla*_KPC-2_ *, bla*_TEM-198_ *, bla*_OXA-1_ *, bla*_OXA-9_ *, bla*_CTX-M-15_ *, oqxA, dfrA14, sulII, ampH*		IncFIB(pQil)_1_pQil_JN233705, IncFIB(K)_1_Kpn3_JN233704
sk140y140t	25 February 2016	17				*oqxBgb, bla*_KPC-2_ *, bla*_TEM-122_ *, bla*_OXA-9_ *, oqxA, ampH*		IncFIB(pQil)_1_pQil_JN233705, IncFIB(K)_1_Kpn3_JN233704
sk141y141t	25 February 2016	35				*oqxBgb, bla*_KPC-2_ *, bla*_TEM-122_ *, bla*_OXA-9_ *, oqxA, tetD, ampH*		IncFIB(pQil)_1_pQil_JN233705, IncFIB(K)_1_Kpn3_JN233704
sk142y142t	22 March 2016	2279				*strA, strB, oqxBgb, bla*_KPC-2_ *, bla*_TEM-198_ *, bla*_OXA-9_ *, aac(3)-Iia, bla*_CTX-M-15_ *, oqxA, dfrA14, sulII, ampH*		IncFIB(pQil)_1_pQil_JN233705, IncFIB(K)_1_Kpn3_JN233704
sk143y143t	31 March 2016	17				*oqxBgb, bla*_KPC-2_ *, bla*_TEM-122_ *, bla*_OXA-9_ *, oqxA, ampH*		IncFIB(pQil)_1_pQil_JN233705, IncFIB(K)_1_Kpn3_JN233704

## Data Availability

The genome assemblies of the sequenced strains are available at the European Nucleotide Archive (ENA). The ID code for each genome submitted is reported in Supplementary Table S3.

## Supplementary Materials

The following are available online at https://www.mdpi.com/article/10.3390/antibiotics10060718/s1, Figure S1. Local prevalence of KPC-Kp and KPC-Ec in the period 2014–2016 in the hospital setting. Table S1. *Escherichia coli* (KPC-Ec) isolates selected for WGS analysis and co-isolation of *Klebsiella pneumoniae* (KPC-Kp) isolates from the same patients. Table S2. *Klebsiella pneumoniae* (KPC-Kp) isolates selected for WGS analysis and co-isolation of *Escherichia coli* (KPC-Ec) isolates from the same patients. Table S3. List of the ENA codes for the genome assemblies of the studied strains.
