# Supplementary material for: Whole-Genome Sequencing Investigation of a Large Nosocomial Outbreak Caused by ST131 H30Rx KPC-Producing Escherichia coli in Italy
Source: Antibiotics (Basel). 2021 Jun 15;10(6):718. doi: 10.3390/antibiotics10060718 (PMC8232337; doi:10.3390/antibiotics10060718)
Supplement: Supplementary file 1 [file antibiotics-10-00718-s001.zip › antibiotics-1219459-supplementary.pdf]

antibiotics

MDPI

Article

Whole-genome Sequencing Investigation of a Large Nosocomial Outbreak Caused by ST131 H30Rx KPC-Producing *Escherichia coli* in Italy

Aurora Piazza [1,*,†], Luigi Principe [2,†], Francesco Comandatore [3,†], Matteo Perini [3,†], Elisa Meroni [4,†], Vittoria Mattioni Marchetti [5], Roberta Migliavacca [1] and Francesco Luzzaro [4]

1 Clinical-Surgical, Diagnostic and Pediatric Sciences Department, Unit of Microbiology and Clinical Microbiology, University of Pavia, 27100 Pavia, Italy; roberta.migliavacca@unipv.it
2 Clinical Pathology and Microbiology Unit, S. Giovanni di Dio Hospital, 88900 Crotone, Italy; luigi.principe@gmail.com
3 Romeo and Enrica Invernizzi Pediatric Research Center, Department of Biomedical and Clinical Sciences L. Sacco, University of Milan, 20157 Milan, Italy; francesco.comandatore@unimi.it (F.C.); matteo.perini@unimi.it (M.P.)
4 Microbiology and Virology Unit, A. Manzoni Hospital, 23900 Lecco, Italy; el.meroni@asst-lecco.it (E.M.); f.luzzaro@asst-lecco.it (F.L.)
5 Biomedical Center, Faculty of Medicine in Pilsen, Charles University,323 00 Pilsen, Czech Republic; vittoria.mattionimarche01@universitadipavia.it
* Correspondence: aurora.piazza@unipv.it
† These authors contributed equally to this work.

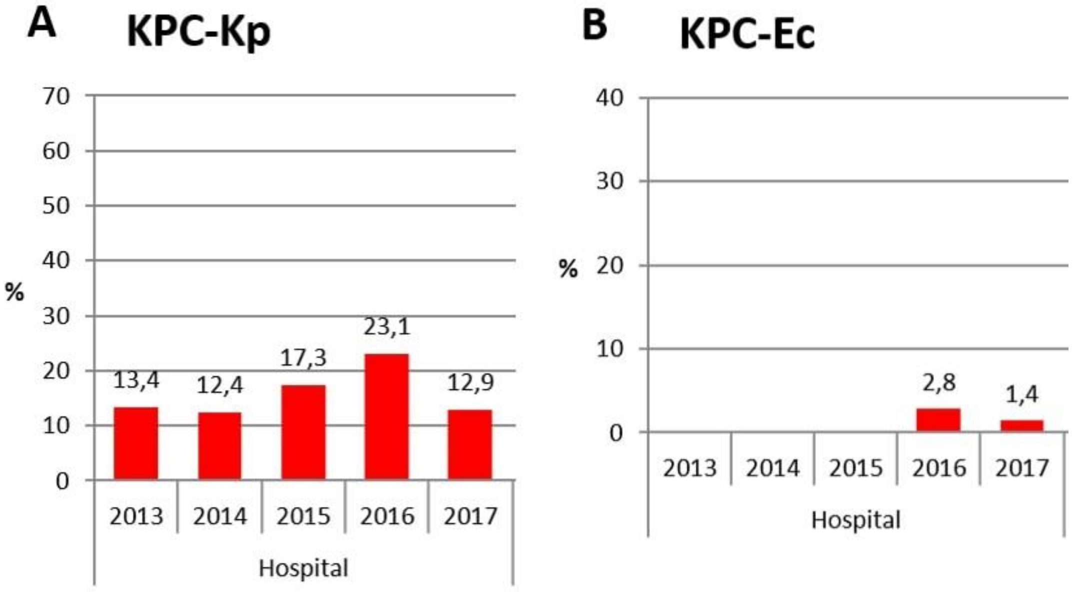

Figure S1. Local prevalence of KPC-Kp and KPC-Ec in the period 2014–2016 in the hospital setting.

Table S1. *Escherichia coli* (KPC-Ec) isolates selected for WGS analysis and co-isolation of *Klebsiella pneumoniae* (KPC-Kp) isolates from the same patients.

ID Sample	KPC-Ec Isolation Date	KPC-Ec—Clinical Sample	KPC-Kp Isolation Date	KPC-Kp—Clinical Sample
sk35y35t	15 February 2016	Rectal swab	28 October 2015	Ulcer
**sk36y36t**	16 February 2016	Rectal swab	15 July 2015	Rectal swab
sk37y37t	26 February 2016	Urine	-	-

sk38y38t	29 February 2016	Rectal swab	08 March 2016	Rectal swab
sk39y39t	01 March 2016	Urine	-	-
sk40y40t	03 March 2016	Purulent exudate	15 July 2015	Rectal swab
sk42y42t	03 March 2016	Urine	31 March 2016	Rectal swab
sk41y41t	08 March 2016	Blood	08 March 2016	Rectal swab
sk43y43t	17 March 2016	Rectal swab	08 March 2016	Rectal swab
sk44y44t	23 March 2016	Blood	08 March 2016	Rectal swab
sk45y45t	05 April 2016	Urine	29 March 2016	Rectal swab
sk46y46t	18 April 2016	Bronchial aspirate	16 February 2016	Rectal swab
sk47y47t	18 April 2016	Urine	-	-
sk48y48t	21 April 2016	Surgical wound swab	16 October 2017	Rectal swab
sk49y49t	24 April 2016	Peritoneal fluid	11 January 2016	Rectal swab
sk50y50t	10 May 2016	Respiratory secretion	-	-
sk51y51t	13 May 2016	Urine	-	-
sk52y52t	22 May 2016	Urine	-	-
sk53y53t	11 June 2016	Urine	19 February 2016	Rectal swab
sk54y54t	25 June 2016	Urine	03 March 2016	Rectal swab
sk55y55t	29 June 2016	Drainage fluid	16 March 2016	Rectal swab
sk56y56t	30 June 2016	Urine	11 February 2016	Rectal swab
sk57y57t	13 July 2016	Urine	02 March 2016	Rectal swab
sk58y58t	13 July 2016	Rectal swab	02 March 2016	Rectal swab
sk59y59t	14 July 2016	Purulent exudate	-	-
sk60y60t	30 September 2016	Rectal swab	16 March 2016	Rectal swab
sk185y185t	14 November 2016	Respiratory secretion	21 July 2015	Rectal swab
sk136y136t	23 January 2017	Rectal swab	-	-
sk137y137t	05 June 2017	Rectal swab	-	-

The index strain is indicated in bold.

Table S2. *Klebsiella pneumoniae* (KPC-Kp) isolates selected for WGS analysis and co-isolation of *Escherichia coli* (KPC-Ec) isolates from the same patients.

ID sample	KPC-Kp—Isolation Date	KPC-Kp—Clinical Sample	KPC-Ec -Isolation Date	KPC-Ec—Clinical Sample
sk138y138t	22 January 2016	Urine	03 March 2016	Purulent exudate
sk139y139t	16 February 2016	Rectal swab	16 February 2016	Rectal swab
sk140y140t	25 February 2016	Rectal swab	25 February 2016	Rectal swab
sk141y141t	25 February 2016	Urine	29 February 2016	Rectal swab
sk142y142t	22 March 2016	Rectal swab	01 March 2016	Rectal swab
sk143y143t	31 March 2016	Rectal swab	03 March 2016	Rectal swab

Table S3. List of the ENA codes for the genome assemblies of the studied strains.

ID	Sample	Experiment	Run
sk35y35t	ERX4530492	ERS5073698 (SAMEA7315155)	ERR4596830
sk36y36t	ERX4530493	ERS5073699 (SAMEA7315156)	ERR4596831
sk37y37t	ERX4530494	ERS5073700 (SAMEA7315157)	ERR4596832
sk38y38t	ERX4530495	ERS5073701 (SAMEA7315158)	ERR4596833
sk39y39t	ERX4530496	ERS5073702 (SAMEA7315159)	ERR4596834
sk40y40t	ERX4530497	ERS5073703 (SAMEA7315160)	ERR4596835
sk42y42t	ERX4530498	ERS5073704 (SAMEA7315161)	ERR4596836
sk41y41t	ERX4530499	ERS5073705 (SAMEA7315162)	ERR4596837
sk43y43t	ERX4530500	ERS5073706 (SAMEA7315163)	ERR4596838
sk44y44t	ERX4530501	ERS5073707 (SAMEA7315164)	ERR4596839
sk45y45t	ERX4530502	ERS5073708 (SAMEA7315165)	ERR4596840

sk46y46t	ERX4530503	ERS5073709 (SAMEA7315166)	ERR4596841
sk47y47t	ERX4530504	ERS5073710 (SAMEA7315167)	ERR4596842
sk48y48t	ERX4530505	ERS5073711 (SAMEA7315169)	ERR4596843
sk49y49t	ERX4530506	ERS5073712 (SAMEA7315170)	ERR4596844
sk50y50t	ERX4530507	ERS5073713 (SAMEA7315171)	ERR4596845
sk51y51t	ERX4530508	ERS5073714 (SAMEA7315172)	ERR4596846
sk52y52t	ERX4530509	ERS5073715 (SAMEA7315173)	ERR4596847
sk53y53t	ERX4530510	ERS5073716 (SAMEA7315174)	ERR4596848
sk54y54t	ERX4530511	ERS5073717 (SAMEA7315175)	ERR4596849
sk55y55t	ERX4530512	ERS5073718 (SAMEA7315176)	ERR4596850
sk56y56t	ERX4530513	ERS5073719 (SAMEA7315177)	ERR4596851
sk57y57t	ERX4530514	ERS5073720 (SAMEA7315178)	ERR4596852
sk58y58t	ERX4530515	ERS5073721 (SAMEA7315179)	ERR4596853
sk59y59t	ERX4530516	ERS5073722 (SAMEA7315180)	ERR4596854
sk60y60t	ERX4530517	ERS5073723 (SAMEA7315181)	ERR4596855
sk185y185t	ERX4530518	ERS5073724 (SAMEA7315182)	ERR4596856
sk136y136t	ERX4530519	ERS5073725 (SAMEA7315183)	ERR4596857
sk137y137t	ERX4530520	ERS5073726 (SAMEA7315184)	ERR4596858
sk138y138t	ERX4530521	ERS5073727 (SAMEA7315185)	ERR4596859
sk139y139t	ERX4530522	ERS5073728 (SAMEA7315186)	ERR4596860
sk140y140t	ERX4530523	ERS5073729 (SAMEA7315187)	ERR4596861
sk141y141t	ERX4530524	ERS5073730 (SAMEA7315188)	ERR4596862
sk142y142t	ERX4530525	ERS5073731 (SAMEA7315189)	ERR4596863
sk143y143t	ERX4530526	ERS5073732 (SAMEA7315190)	ERR4596864
